# WHO's health inequality data repository

**DOI:** 10.2471/BLT.23.290004

**Published:** 2023-05-01

**Authors:** Ahmad Reza Hosseinpoor, Nicole Bergen, Katherine Kirkby, Anne Schlotheuber, Daniel A Antiporta, Stephen Mac Feely

**Affiliations:** a Division of Data, Analytics and Delivery for Impact, World Health Organization, Avenue Appia 20, 1211 Geneva 27, Switzerland.

As the World Health Organization (WHO) marks its 75th anniversary year, it remains steadfast in its pledge to address social inequalities and advance equity in health. WHO is committed to achieving the sustainable development goals (SDGs), which are founded on the promise to leave no one behind. Global health agencies acknowledge the importance of tackling inequality as they strive to end human immunodeficiency virus (HIV) infections;^1^ strengthen health emergency preparedness, response and resilience:^2^ expand the coverage of childhood immunizations;^3^ and accelerate improvements in women’s, children’s and adolescent’s health,^4^ among other areas. Likewise, health equity is stated as a goal across many national and subnational health policies and plans, as well as global health and development initiatives.

Disaggregated data – which show how people of different ages, sex, economic status, education levels, place of residence and other characteristics, experience health or other conditions – are a vital part of advancing equity. Disaggregated data serve as a basis for measuring and monitoring health inequalities and developing evidence-informed solutions to tackle unfair situations. Routine health inequality monitoring is a mechanism for evaluating progress towards equity goals and targets and ensuring accountability. Yet, evidence suggests that the importance of disaggregated health data is not fully appreciated. A recent WHO survey found that only half of countries included disaggregated data in their published national health statistics reports.^5^

Launched in April 2023 by WHO, the health inequality data repository aims to make disaggregated data more accessible to diverse global audiences, including policy-makers, analysts, researchers, health professionals and others.^6^ The repository contains the largest collection of publicly available disaggregated data on health and health-relevant topics, with about 11 million data points. The repository includes over 2000 indicators related to the SDGs; coronavirus disease 2019 (COVID-19); reproductive, maternal, newborn and child health; immunization; HIV, tuberculosis and malaria; adult health; health care; burden of disease; disability; environmental health; WHO Thirteenth General Programme of Work; and other health determinants. Indicators are disaggregated by one or more dimension of inequality, according to relevancy and data availability. Available dimensions of inequality include: age, birth order, city, disability, economic status, education, employment status, employment type, ethnicity, race or caste, health worker status, marital status, migratory status, number of living children, place of residence, religion, sex and subnational region. Drawn from over 15 sources, the data cover diverse populations from all world regions, dating from 1950 to present. Disaggregated data were sourced from existing global, regional and multicountry databases following a systematic scoping exercise, and data were prepared in a standard format. Data sets will be added and/or updated as new data become available. Statistical quality standards are employed to ensure that data sets included in the repository are timely, complete, accurate and reliable.

A key feature of the repository is its user-friendly interface, which makes it accessible to audiences with a range of technical skills. The repository allows users to explore data through an interactive data visualization software that was specifically developed for health inequality analyses, the health equity assessment toolkit.^7^ Through the toolkit, users can dynamically customize the data on display and choose from a variety of graphs and tables. The toolkit also facilitates calculating summary measures of inequality, benchmarking between settings, and generating and exporting visual outputs. All data sets and accompanying metadata are available for download and are accessible through an application programming interface.

The repository represents an important contribution to global public health and will be a resource for global efforts to monitor inequalities and advance health equity. For instance, the repository brings together disaggregated data for all available SDG indicators, thereby supporting the use of data to understand where progress has been made equitably and where more action is needed. Analysis of disaggregated data on COVID-19 is helping to reveal lessons for equity-oriented pandemic response and recovery. In the area of HIV, tuberculosis and malaria, disaggregated data were the basis for the global report *State of inequality: HIV, tuberculosis and malaria*,^8^ which demonstrated widespread inequalities across these diseases and created awareness to drive action on unfair inequalities.

The repository also opens new opportunities for expanded inequality monitoring. In-depth explorations of inequalities will bolster important ongoing work to achieve improved health, living conditions and well-being among disadvantaged population groups. WHO has developed resources to build capacity for health inequality monitoring across diverse applications.^9^

The impact and relevance of the Health Inequality Data Repository require the sustained and expanded collection of high-quality disaggregated data pertaining to diverse topics, settings and populations. Widespread and dedicated efforts to use disaggregated data to the fullest extent possible to advance health equity are also needed. 
